# Epidermal Growth Factor Receptor (EGFR) Gene Mutation Analysis in Adenocarcinoma of Lung, the First Report from Iran

**DOI:** 10.30699/IJP.14.1.1

**Published:** 2018-12-27

**Authors:** Sahand Mohammadzadeh, Zahra Jowkar, Mitra Mirzai, Bita Geramizadeh

**Affiliations:** 1 *MD, Assistant Professor of Pathology, Dept. of Pathology, Medical School of Shiraz University, Shiraz University of Medical Sciences, Shiraz, Iran*; 2 *MS, Dept. of Pathology, Medical School of Shiraz University, Shiraz University of Medical Sciences, Shiraz, Iran*; 3 *MSc, Dept. of Pathology, Medical School of Shiraz University, Shiraz University of Medical Sciences, Shiraz, Iran*; 4 *MD, Professor of Pathology, Medical School of Shiraz University , Shiraz University of Medical Sciences, Shiraz, Iran*; 5 *Transplant Research Center, Shiraz University of Medical Sciences, Shiraz, Iran*

**Keywords:** EGFR Genes, Mutation, Lung cancer, Iranian

## Abstract

**Background and Objective:**

Epidermal growth factor receptor (EGFR) gene mutation, especially in exons 18 to 21, is an important predictor of the response rate of lung adenocarcinoma to tyrosine kinase inhibitors. There are variable reports from Asian and European countries, as well as North America, about the frequency of the EGFR mutation in lung adenocarcinoma, yet molecular study about this incidence has been published from Iran. In this study, we investigated the frequency of this mutation in our center, which is the largest referral center in the south of country. This report will be the first published article about EGFR mutational analysis from Iran.

**Methods:**

During the study period (September 2011 till September 2016) i.e. 5 years, there have been 50 cases of pathologically-confirmed lung adenocarcinoma. These cases underwent mutational analysis for exons 18 to 21 of the EGFR gene by PCR and DNA sequencing. All demographic findings were also extracted from the patients’ charts and recorded.

**Results:**

There were 30 male and 20 female patients, with an average age of 58 years. The overall frequency of EGFR mutation was 28% (14 out of 50). The most common mutation was Del 19 (10 of 14, 71.4%), 3 mutations were found in exon 20 and one mutation was found in exon 21. EGFR mutations were more frequent in women than in men (30% versus 26.7%) and in nonsmokers than in smokers (37.9% versus 14.3%).

**Conclusion:**

Lung adenocarcinoma with EGFR mutation shows strong association with female non-smokers. Our results showed an intermediate frequency of this mutation, which was higher than results from Western countries and lower than most Asian countries.

## Introduction

Lung adenocarcinoma is one of the common lung cancers found in humans and is a common cause of cancer death in both men and women (1). EGFR is a transmembrane receptor tyrosine kinase protein of the ErbB family that is expressed in some epithelial tissues (2). The binding of a ligand to this receptor activates its tyrosine kinase activity, resulting in the dimerization of EGFR molecules and phosphorylates several substrates on a number of signal transduction pathways, leading to cell proliferation, DNA synthesis and cell growth. The over- expression of EGFR has been reported in the pathogenesis of many human malignancies including non-small cell lung cancer (NSCLC) (3, 4).

It has been shown that a subset of patients with lung adenocarcinoma (10 to 40%) have specific active EGFR mutations, mostly in exons 18 to 21, that are associated with increased sensitivity to tyrosine-kinase inhibitors (TKIs) such as gefitinib (Iressa) or erlotinib (Tarceva). (5). Therefore recent guidelines suggest that EGFR mo- lecular testing and mutation analysis should be performed in all patients with lung adenocarcinoma (6). There has been one study from Mashhad (Iran) on the Immunohistochemical expression of EGFR in lung cancer (7), however, to the best of our knowledge there has been no report on the EGFR mutation’s molecular analysis and incidence in lung adenocarcinoma from Iran, so in this study we attempt to evaluate the prevalence of these muta- tions in our center, the largest referral center in the south of Iran.

## Materials and Methods

In this 5 year-long study (September 2011 to September 2016), we collected 50 cases of confirmed lung adeno- carcinomas (40 biopsies and 10 resection samples) from the pathology archives in hospitals affiliated with the Shiraz University of Medical Sciences.

The H&E slides were reviewed by the pathologist to confirm the adequacy of tissue specimens and the histo- logic diagnosis of adenocarcinoma. Clinical information such as gender, age, and smoking history were obtained from medical records.

**Figure1:**
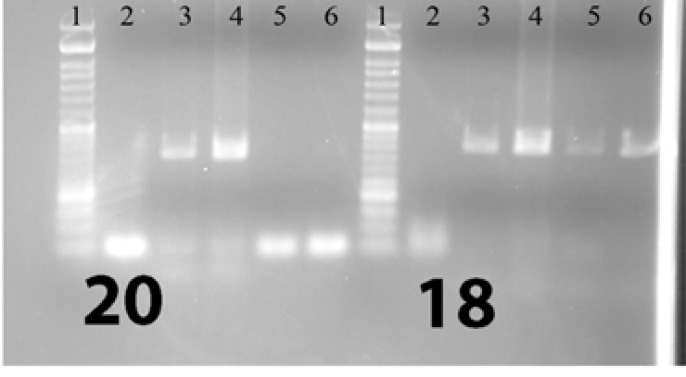
Visualized product on electrophoresis gel for simple PCR (first run) of exons 18 and 20 1: Ladder 2: Negative control 3, 4, 5, 6: Patients

**Figure 2:**
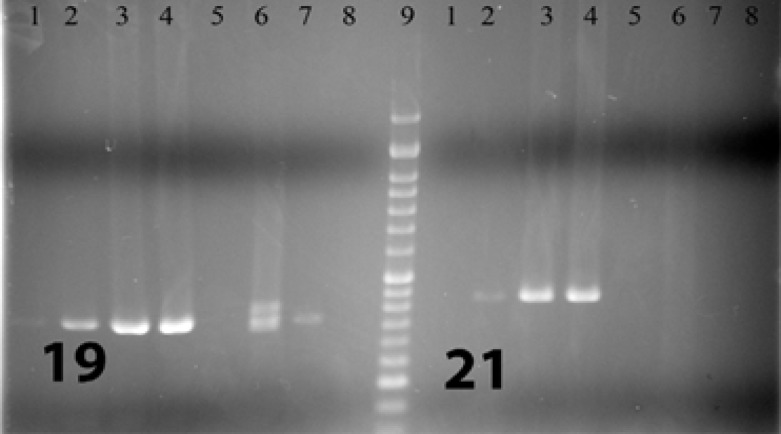
Visualized product on electrophoresis gel for simple PCR (first run) of exons 19 and 21. 1, 2, 3, 4, 5, 6, 7: Patients 8: Negative control 9: Ladder

**Figure 3:**
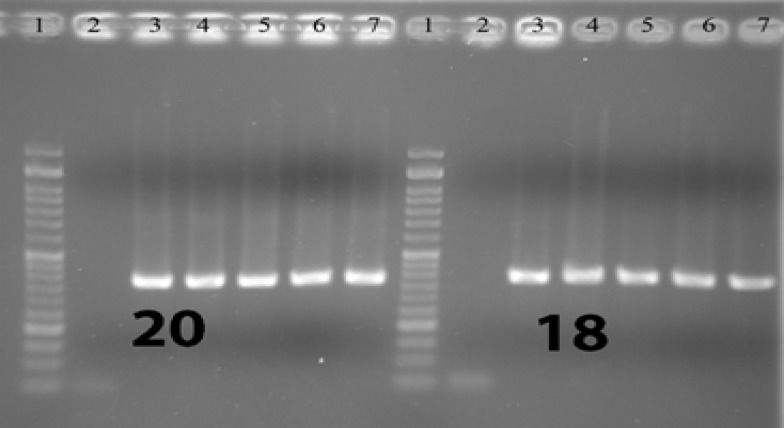
Visualized product on electrophoresis gel for nested PCR of exons 18 and 20. 1: Ladder 2: Negative con- trol 3,4,5,6,7: Patients

**Figure 4:**
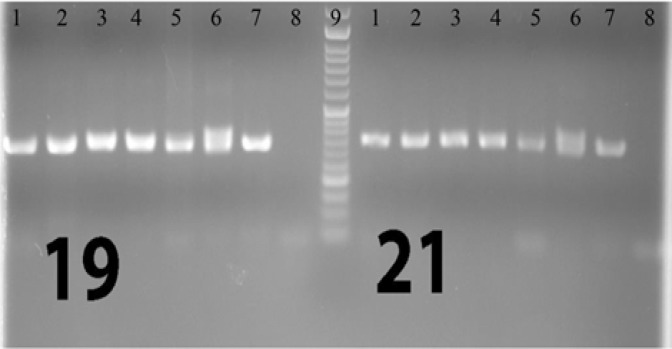
Visualized product on electrophoresis gel for nested PCR of exons 19 and 21

For each case, DNA from formalin-fixed paraffin-embedded tissue (FFPE) was isolated using the Qiagen (USA) DNA extraction kit. After DNA extraction, PCR amplification was performed and then the PCR product sequenced using the by Sanger method. PCRs of exons 18, 19 , 20, and 21 were performed in both sense and antisense directions so as to increase the study’s sensitivity and specifity, and the product of the first step PCR (primary PCR) was used in secondary (nested) PCR. Figures 1-4 show the details of the primary and secondary

1, 2, 3, 4, 5, 6, 7: Patients 8: Negative control 9: Ladder

PCRs. This method was the same as the one used in the study by Lynch TJ et al in 2004 (8). Figures 1 to 4 show the PCRs for each mutation separately.

## Results

Our study included 50 cases of lung adenocarcinomas. Thirty cases were males and 20 cases were females. The average age and age range of the cases were 58.4 ± 13. Among these 50 cases, 29 (58%) cases were non-smokers and 31(42%) were smokers. The overall frequency of the EGFR mutation was found in 28% (14 of 50) of the adenocarcinomas. The most common mutation was Del 19 (10 of 14, 71.4%); 3 mutations were found in exon 20 and one mutation was found in exon 21. The mutation pattern and different characteristics of these 14 patients are summarized in tables 1 and 2.

**Table 1:** Characteristics of 14 cases of lung cancer with EGFR mutation

	**Gender**	**Age**	**Smoking history**	**Exon**	**Mutation type**	**Amino acid Alteration**
1	Female	55	Negative	20	Ins AWT	N771-H773
2	Female	76	Negative	20	Ins PT	V774-C775
3	Female	53	Negative	19	Del	E746-A750
4	Male	37	Negative	19	Del	E746-A750
5	Male	80	Negative	19	Del	E746-A750
6	Male	50	Negative	20	Ins G	E770-N771
7	Male	71	Negative	19	Del	E749-753
8	Female	55	Negative	19	Del	E746-750
9	Female	47	Positive	19	Del	E746-750
10	Male	55	Positive	21	Sub	D855H
11	Female	59	Negative	19	Del	E746-750
12	Male	69	Negative	19	Del	E746-750
13	Male	76	Positive	19	Del	E746-750
14	Male	59	Negative	19	Del	E746-750

**Table 2:** Percentage of mutation in different cases according to clinical characteristics

**Characteristic**	**Numbers**	**EGFR** **Positive (%)**
Men	30	8(26.7%)
Women	20	6(30%)
Smokers	21	3(14.3%)
Non-smokers	29	11(37.9%)
Woman & non-smoker	16	5(31.3%)
Woman & smoker	4	1(25%)
Man & non-smoker	13	6(46.2%)
Men & smoker	17	2(11.8%)
Total	50	14(28%)

EGFR mutations were more frequent in women than in men (30% versus 26.7%) and in non-smokers than in smokers (37.9% versus 14.3% respectively). The frequency of EGFR mutations was 31.3 % (5 of 16) in non- smoking women.

Among the 50 studied cases of lung adenocarcinomas, 27 cases (54%) concerned well-differentiated adenocar- cinomas and 23 (46%) cases were moderately or poorly differentiated adenocarcinomas. EGFR mutations were more frequently found in well differentiated tumors than moderately or poorly differentiated tumors (9 of 27, 33.3% versus 5 of 23, 21.7% respectively), but the P value was not statistically significant (*P*>0.05).

## Discussion

Lung adenocarcinomas harboring EGFR mutations are a distinct biological subset, as evidenced by their high response rate to TKIs. Therefore, the identification of the incidence of EGFR mutations is very important for each geographic region. Since 2010, the evaluation of EGFR mutations in lung adenocarcinoma has been recom- mended as one of the main steps of clinical workups (8, 9). Two EGFR mutations in exons 19 and 21 account for about 90% of all EGFR mutations reported in lung adenocarcinomas, and are the best predictors of the response and survival benefits of EGFR TKIs. Screening for these mutations in patients with lung adenocarcinomas can be used to predict which patients will respond to the EGFR TKIs.

There have been different reported incidences of EGFR mutations in lung adenocarcinomas around, depending on ethnicity, reported to be 40% in East Asia, 15% in Europe, 22% in North America, 26% in the Indian subconti- nent, 36% in South America, 12% in Oceania and 21% in Africa (8, 10, 11, 12, 13). To the best of our knowledge, there has been no report on the incidence of EGFR mutations in lung adenocarcinomas from Iran, and this study is the first one performed in Iran from a molecular approach on the frequency of EGFR mutations and their relation to the clinicopathological characteristics of lung adenocarcinomas. In our study, in the largest referral center in the South of Iran, the prevalence of EGFR mutations in lung adenocarcinomas was 28%. The frequency of mutations was lower compared with East Asian countries and higher than European and African countries (13).

Despite variable reports from different geographic locations around the world, approximately 45-54% of re- ported mutations concerned inframe deletions in exon 19, 40% were missense mutations in L858R in exon 21, and 4-9 % of mutations were reported in exons 18 and 20 (10). In our study, the most frequent mutation was an inframe deletion in exon 19, which comprised up to 71.4% of all mutations. The lower frequency of exon 21 mutations in our study (2%), compared with other results, is probably due to racial differences. However, the incidence of mutations in exons 18 and 20 were also low in our study, as in previous studies. It must be noted that two major EGFR-TKIs resistant mutations (exon 20 insertions and T790M) were not found in our study (10-13).

In 2016, a systematic review and meta-analysis was done to detect the prevalence of EGFR mutation in patients with non-small cell lung cancer by Zhang, et al. In this study a total of 456 studies were included, reporting 30,466 patients with EGFR mutations among 115,815 NSCLC patients. The overall prevalence for EGFR muta- tions was 32.3%, ranging from 38.4% in China to 14.1% in Europe. The overall prevalence of EGFR mutations was higher in females (females vs. males: 43.7% vs. 24.0%) and non-smokers (non-smokers vs. smokers: 49.3% vs. 21.5%) (14).

Gender is one of the most important and known clinical predictors for Epidermal Growth Factor Receptor mu- tations, and the female gender appears to be more susceptible to these mutations both in our study and previous reports (Asian and European) (Sun P-L et al , 2010). Among Asian reports, the overall reported EGFR mutation frequency in lung adenocarcinomas were 60% in females and 37% in males. In European countries, the overall EGFR mutation frequency was 9% in males and 22% in females. In North America, a 19% EGFR mutation fre- quency was detected in males, compared to 28% in females, and in Africa this frequency was 8% in men and 48 % in women (10-14).

In our study, EGFR mutations were more frequent in women than in men (30% versus 26.7%) and in non- smokers than in smokers (37.9% versus 14.3%), but the P value was not statistically significant. The frequency of EGFR mutations was 31.3% (5 out of 16) in non-smoking women in our study. Previous studies have shown that EGFR mutations are more frequent in non-smokers than in smokers (63.4% versus 32.0%) and frequency of EGFR mutations was 68% in non-smoking women (11).

One of the reasons for the relatively lower rates of EGFR mutations in smokers has been explained with more prevalent K-ras mutations in this group of patients. Since K-ras and EGFR mutations were mutually exclusive, EGFR mutations are less frequent in smokers (12), and smoker tumors frequently exhibit K-ras mutations (a downstream effector in the EGFR pathway). So in non-smokers, the EGFR signaling pathway can be selectively activated and can be mutated in many cases (12, 13).

In a meta-analysis study in 2015 by Midha in lung adenocarcinomas, the overall EGFR mutation frequency in the Asian Pacific region was 64% in non-smokers against 33% in smokers, and this result was obtained by reviewing 20 studies. In Europe, the result was 35% in non-smokers and 8% in smokers (10).

In our 50 cases of adenocarcinomas, 27 cases (54%) were well-differentiated adenocarcinomas and 23 cases were (46%) moderately or poorly differentiated adenocarcinomas. EGFR mutations were more frequently found in welldifferentiated tumors than moderately or poorly differentiated tumors (9 of 27, 33.3% versus 5 of 23, and 21.7% respectively). This finding is similar to previous studies (14-17).

## Conclusion

As a conclusion, lung adenocarcinomas with EGFR mutations is a distinct biological subset, as evidenced by its strong association with the female and non-smoker groups. Our results showed an intermediate frequency of this mutation, which was higher than Western countries and lower than most Asian countries.

## References

[1] Coate LE, John T, Tsao M-S, Shepherd FA (2009). Molecular predictive and prognostic markers in non-small-cell lung can- cer. The Lancet Oncol.

[2] Sholl LM, Yeap BY, Iafrate AJ, Holmes-Tisch AJ, Chou Y-P, Wu M-T (2009). Lung adenocarcinoma with EGFR am- plification has distinct clinicopathologic and molecular features in never-smokers. Cancer Res.

[3] Paez JG, Jänne PA, Lee JC, Tracy S, Greulich H, Gabriel S (2004). EGFR mutations in lung cancer: correlation with clinical response to gefitinib therapy. Science.

[4] Bethune G, Bethune D, Ridgway N, Xu Z (2011). Epidermal growth factor receptor (EGFR) in lung cancer: an overview and update. Journal Thorac Dis.

[5] Liang Z, Zhang J, Zeng X, Gao J, Wu S, Liu T (2010). Relationship between EGFR expression, copy number and mutation in lung adenocarcinomas. BMC cancer.

[6] Lindeman NI, Cagle PT, Beasley MB, Chitale DA, Dacic S, Giaccone G (2013). Molecular testing guideline for selec- tion of lung cancer patients for EGFR and ALK tyrosine kinase inhibitors: guideline from the College of American Pathologists, International Association for the Study of Lung Cancer, and Association for Molecular Pathology. J Thorac Oncol.

[7] Basiri R, Jafarian AH, Karimi M, Mohammadzadeh Lari Sh, Haghgoo SM (2015). Expression of Epidermal Growth Factor Receptor and the association with Demographic and Prognostic Factors in Patients with Non-small Cell Lung Cancer. J Cardiothorac Med.

[8] Lynch TJ, Bell DW, Sordella R, Gurubhagavatula S, Okimoto RA, Brannigan BW (2004). Activating mutations in the epidermal growth factor receptor underlying responsiveness of non-small cell lung cancer to Gefitinib. N Engl J Med.

[9] ChoiY-L, SunJ-, ChoJ, RampalS, HanJ, ParasuramanB, etal (2013). EGFRmutationtestinginpatientswithadvancednon-small celllungcancer:acomprehensiveevaluationofreal-worldpracticeinanEastAsiantertiaryhospital. PLoSOne.

[10] Midha A, Dearden S, McCormack R (2015). EGFR mutation incidence in non-small-cell lung cancer of adenocarcino- ma histology: a systematic review and global map by ethnicity (mutMapII). Am J of cancer Res.

[11] Sun P-L, Seol H, Lee HJ, Yoo SB, Kim H, Xu X (2012). High incidence of EGFR mutations in Korean men smokers with no intratumoral heterogeneity of lung adenocarcinomas: correlation with histologic subtypes, EGFR/TTF-1 expressions, and clinical features. J Thorac Oncol.

[12] Unal OU, Oztop I, Calibasi G, Baskin Y, Koca D, Demir N (2013). Relationship between epidermal growth factor re- ceptor gene mutations and clinicopathological features in patients with non-small cell lung cancer in western Turkey. Asian Pac J Cancer Prev.

[13] Gahr S, Stoehr R, Geissinger E, Ficker J, Brueckl W, Gschwendtner A (2013). EGFR mutational status in a large series of Caucasian European NSCLC patients: data from daily practice. Br J of cancer.

[14] Zhang Y-L, Yuan J-Q, Wang K-F, Fu X-H, Han X-R, Threapleton D (2016). The prevalence of EGFR mutation in patients with non-small cell lung cancer: a systematic review and meta-analysis. Oncotarget.

[15] Li AR, Chitale D, Riely GJ, Pao W, Miller VA, Zakowski MF (2008). EGFR mutations in lung adenocarcinomas: clini- cal testing experience and relationship to EGFR gene copy number and immunohistochemical expression. J of Mol Diag.

[16] Ren JH, He WS, Yan GL, Jin M, Yang KY, Wu G (2012). EGFR mutations in non‐small‐cell lung cancer among smokers and non‐smokers: A meta‐analysis. Environ and Mol mutagen.

[17] Tomizawa Y, Iijima H, Sunaga N, Sato K, Takise A, Otani Y (2005). Clinicopathologic significance of the muta- tions of the epidermal growth factor receptor gene in patients with non–small cell lung cancer. Clin Cancer Res.

